# Wnt5a Does Not Support Hematopoiesis in Stroma-Free, Serum-Free Cultures

**DOI:** 10.1371/journal.pone.0053669

**Published:** 2013-01-14

**Authors:** Aneta M. Schaap-Oziemlak, Sarah Schouteden, Satish Khurana, Catherine M. Verfaillie

**Affiliations:** Interdepartementeel Stamcelinstituut, Katholieke Universiteit Leuven, Leuven, Belgium; French Blood Institute, France

## Abstract

Previously we reported that Wnt5a is highly expressed in the murine urogenital ridge-derived UG26-1B6 cells but not embryonic liver-derived EL08-1D2 cells. Mouse long-term repopulating hematopoietic stem cells (LTR-HSC) were maintained in non-contact UG26-1B6 cultures but not EL08-1D2 non-contact cultures, unless Wnt5a was also added to the cultures, suggesting a role for Wnt5a in the *in vitro* maintenance of LTR-HSC. Here, we investigated if the effect of Wnt5a on adult LTR-HSC activity is HSC-autonomous. To test the effect of Wnt5a on maintenance of LTR-HSC, we performed limiting dilution competitive transplantation assays of murine Lin-Sca1^+^ c-kit^+^ (LSK) cells cultured for 5 days with TPO and SCF with and without Wnt5a. The effect of Wnt5a on the generation of colony forming units (CFU) and the homing ability of LSK progeny was also tested. No effects were found of Wnt5a on total cell expansion, the number of CFU, or homing ability of day 5 LSK progeny. Furthermore, addition of Wnt5a did not improve, but may have impeded maintenance of LTR-HSC. In conclusion, our data indicate that Wnt5a does not enhance the maintenance and expansion of adult murine LTR-HSCs or committed progenitors cultured *in vitro* in serum- and stroma-free conditions.

## Introduction

Wnt proteins are a large family of secreted glycoproteins that can affect cell fate decisions in many cell compartments. Depending on the Wnt protein, the cell type, the type of receptor, the availability of intracellular signalling molecules, signalling from Wnts occurs via the canonical or non-canonical pathways. In the canonical pathway, binding of Wnt to the Fzd/LRP receptor complex results in stabilization of activated β-catenin, which translocates to the nucleus where it interacts with T Cell Specific Factor and Lymphoid Enhancer Factor (LEF/TCF), and serves as a transcription factor. The non-canonical pathways do not stabilize β-catenin, but cause increases in intracellular Ca^2+^ levels or activate Jun N-terminal Kinase (JNK) (reviewed in [1]), leading to for instance actin-dependent cytoskeleton reorganisation (reviewed in [2]).

Multiple Wnt proteins and their receptors have been identified in most hematopoietic sites during mouse development at the time of detectable hematopoietic stem cell (HSC) activity [3]–[6]. Functional studies of Wnts in murine HSC biology have focused chiefly on Wnt3a, and to a lesser extent on Wnt5a and Wnt10a [7], [8]. Wnt3a-deficiency leads to irreversible and significantly impaired repopulation potential of murine fetal liver (FL) HSC [9]. As Wnt3a deficiency causes embryonic lethality, HSC in postnatal Wnt3a^−/−^ mice cannot be studied [10]. However, elimination of β-catenin alone or combined with γ-catenin in postnatal hematopoietic cells does not lead to impairment in hematopoiesis, suggesting that canonical Wnt signalling does not affect postnatal HSC [11], [12]. Similarly, due to perinatal lethality of Wnt5a^−/−^ mice, the *in vivo* role of Wnt5a in maintenance of adult HSC cannot be studied [13].

Another method to assess the effect of Wnts on HSC is by *ex vivo* culture of HSC in the presence of Wnts and assessing the repopulating and differentiation ability of the HSC. Most of these studies have evaluated the role of Wnts in complex culture systems, which contained stromal cells, medium conditioned by stromal cells or fetal calf serum [14], [15]. For instance, Wnt3a was reported to enhance the repopulating ability of HSC derived from Bcl2^−/−^ mice cultured in serum containing conditions [15]. However, when similar studies were done using serum-free culture conditions, the repopulation ability of Wnt3a treated cells was decreased [7], and when Wnt3a was presented in the context of feeders, no evidence for HSC expansion was found [14]. Several studies have also assessed the effect of Wnt5a on HSC. Culture of HSC on stromal cells overexpressing Wnt5a affects the commitment of HSC to the B-cell lineage [14]. When HSC were cultured in serum-free, stroma-free cultures either alone or supplemented with Stem Cell Factor (SCF) and Fetal Liver Tyrosine Kinase 3 Ligand (Flt3L), HSC quiescence was induced which was accompanied with enhanced repopulation ability of the cultured progeny [7]. The latter was believed to be via non-canonical signalling pathways. From these studies, it is clear that the effects of Wnts appear to be highly influenced by the conditions via which they are presented to HSC.

We previously reported that Wnt5a is highly expressed in the urogenital ridge derived UG26-1B6 cell line but not the embryonic liver derived EL08-1D2 cell line [16]. HSC were maintained in non-contact UG26-1B6 cultures but not EL08-1D2 non-contact cultures, unless Wnt5a was also added to the culture system, suggesting a role for Wnt5a in the *in vitro* maintenance of HSC [16]. To address whether the effect of Wnt5a was on the HSC themselves, we here aimed to examine the effect of Wnt5a in serum-free and stroma-independent conditions on adult murine hematopoietic progenitors and LTR-HSC. Our results show that soluble Wnt5a does not improve the maintenance of adult murine LTR-HSCs, able to self-renew or committed progenitors cultured *in vitro* in serum- and stroma-free conditions.

## Materials and Methods

All animals were purchased from Charles-Rivers Jackson Laboratories (Wilmington, MA) and maintained under SPF (specific pathogen free) conditions in the animal facility of the Katholieke Universiteit Leuven. All mice were sacrificed by cervical dislocation. Regarding transplantation experiments recipient mice were lethally irradiated with a single dose of 8,5 Gy (TBI). All the transplanted mice were monitored twice a week where close behavioral observations of any symptoms of infections were carried out. In addition, in order to prevent any infections all the transplanted mice were maintained on Baytril (entroflaxin; Bayer Health Care, Shawnee Mission, KS) drinking water. All studies were approved by the Ethics Committee of the Katholieke Universiteit Leuven.

Isolation of LSK cells, competitive repopulation assays, and CFU-C assays were done using standard methods. Detailed protocols are available online in the **File S1**.

### *In vitro* Culture System

50 LSK cells per well were plated in 96-well U bottom plate (BD Biosciences, San Jose, MA) in 100 µl of serum-free Stemspan medium (Stem Cell Technologies) containing 2% Penicilin/Streptomycin (Gibco Invitrogen) and cytokines (R&D Systems) at following concentrations: recombinant mouse (rm) thrombopoietin (TPO) (100 ng/ml), rm SCF (50 ng/ml), rmWnt5a (100 ng/ml). LSK cultures were performed for 5 days at 37°C and 5% CO_2_.

### *In vivo* Homing Assay

Progeny of 100 LSK cells cultured for 5 days with TS and TSW were injected i.v. into adult irradiated recipients (25000 cells of LSK progeny per recipient). After 16 hrs bone marrow (BM) was harvested, and colony forming units (CFU) enumerated as a measure of homing (protocol adapted from [17]).

### Statistical Analysis

A Student’s two-tail t-test (paired and unpaired) was performed and p≤0.05 was defined as statistically significant. CRU (competitive repopulating unit) frequency was calculated using ELDA software ([18]).

## Results

**Figure 1:**
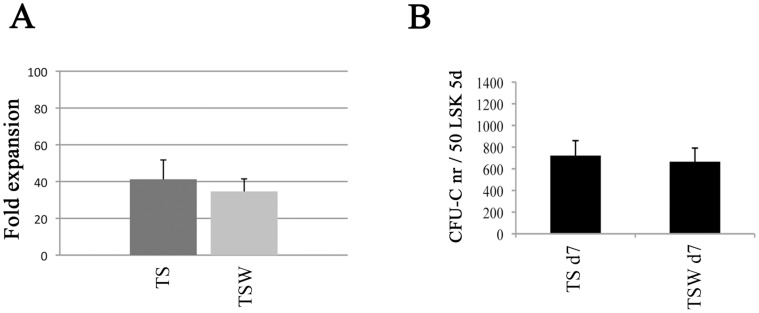
*Ex vivo* expansion of LSK cells with and without Wnt5a. (A) Cell expansion of LSK cells cultured for 5 days with TPO and SCF alone (TS) or with Wnt5a (TSW) (n = 8). (B) CFU-C frequency in progeny of 50 LSK cells cultured for 5 days with TPO and SCF alone (TS) or with Wnt5a (TSW) (n = 8).

Murine BM-derived LSK (Lin- Sca-1^+^ c-kit^+^) cells were cultured for 5 days in the presence of TPO (100 ng/ml) and SCF (50 ng/ml) alone (TS condition), or with Wnt5a (100 ng/ml) (TSW condition). SCF and TPO have been shown previously to maintain murine HSC activity [19], [20]. After 5 days of *in vitro* culture, cells expanded 41-fold and 35-fold in the TS and TSW conditions, respectively **(** **Figure 1A** **)** (p = 0.11), indicating that Wnt5a does not affect LSK cell expansion. We also performed CFU-C assays on TS- and TSW-treated day 5 LSK progeny. The number of CFU-C in progeny of 50 LSK cells was similar for TS- and TSW-treated cells (p = 0.77) **(** **Figure 1B**), demonstrating that Wnt5a does not affect hematopoietic progenitor activity *in vitro*.

**Figure 2:**
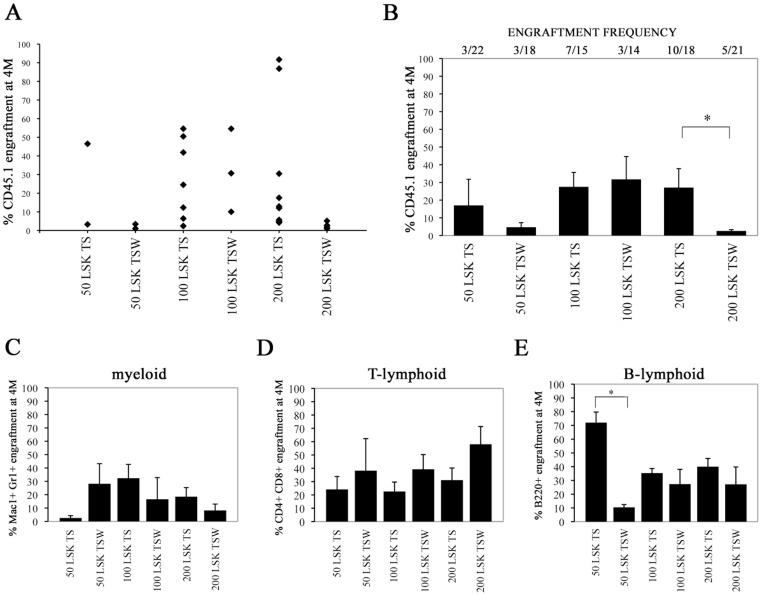
Multilineage engraftment analysis in primary recipients of LSK progeny cells cultured with and without Wnt5a. CD45.2 recipients were transplanted with progeny of 50, 100 or 200 CD45.1 LSK cells cultured for 5 days with TPO and SCF alone (TS) or with Wnt5a (TSW) combined with 10^5^ CD45.2 bone marrow (BM) cells. Multilineage CD45.1 engraftment was evaluated after 4 months (4M) in the peripheral blood (PB) (A) Shown are the percentages of CD45.1^+^ cells in PB at 4M posttransplantation in each primary positively repopulated recipient (≥1% overall donor-derived cells contributing to multilineage engraftment) represented by rhombus in different conditions indicated in the figure. (B) Numbers above the graph represent the number of positively reconstituted (≥1% CD45.1 multilineage engraftment) mice/total number injected mice per group. The graph represents mean with SEM of donor-derived engraftment in primary positively repopulated recipients per group. * = *p≤0.05.* (C, D, E) Mean with SEM donor myeloid (CD11b^+^cells), T-lymphoid (CD4/CD8^+^ cells) and B-lymphoid (B220^+^ cells) chimerism per group. * = *p≤0.05*.

We next tested the effect of Wnt5a on the maintenance of long-term repopulating (LTR)-HSC. We transplanted progeny of 50, 100 and 200 CD45.1 LSK cells cultured for 5 days with TS or TSW together with 1×10^5^ competitor cells (total BM CD45.2 cells) into lethally irradiated adult CD45.2 recipients. Donor-derived (CD45.1) engraftment in the peripheral blood (PB) was measured 4 months after transplantation **(** **Figure 2A, B** **)**. The percent animals repopulated with progeny of 50 LSK cells was similar for the TS or TSW condition. However, the percent animals repopulated with progeny of 100 and 200 LSK cells was 2–3 fold higher for TS than TSW treated cells. Moreover, the levels of donor chimerism were significantly higher for animals that received 200 LSK progeny from cultures with TS compared with cultures with TSW (10.4-fold; p = 0.05). Notably, the CRU frequency was 2.3-fold lower in primary recipients of TSW-treated compared with TS treated grafts (p = 0.0267).

As Malhotra *et al.,* demonstrated that culture of murine HSC on OP9 cells expressing Wnt5a affects B-lymphoid commitment [14], we also evaluated the contribution of TS- and TSW-treated grafts to the different hematopoietic lineages *in vivo*. Because the statistical significance of diminished B-cell repopulation was reached only for 50 LSK dose of TSW-treated cells compared with TS-treated cells, it appears to be that Wnt5a does not overall affect B- and T-lymphoid engraftment of TSW-treated LSK cells **(** **Figure 2C–E** **)**.

**Figure 3:**
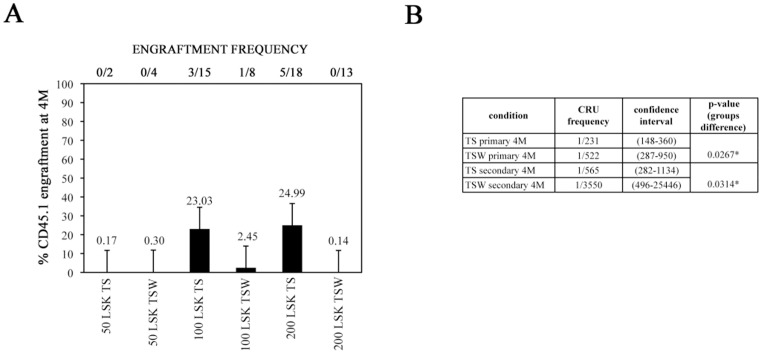
Multilineage engraftment analysis in secondary recipients of LSK progeny cells cultured with and without Wnt5a. One million BM cells from positively repopulated (≥1% CD45.1 multilineage engraftment) primary recipients of LSK cell progeny cultured with TS or TSW were grafted in secondary CD45.2 recipients. Multilineage CD45.1 engraftment was evaluated after 4 months (4 M) in the peripheral blood (PB) (A) Numbers above the graph represent the number of positively reconstituted (≥1% CD45.1 multilineage engraftment) mice/total number mice injected per group. The graph represents mean with SEM of percent chimerism per group. (B) Competitive repopulation unit (CRU) frequency in primary and secondary recipients calculated using ELDA software ([18]); * = *p≤0.05*.

We also performed secondary transplantations by injecting 1×10^6^ total BM cells from each positively engrafted primary recipient into lethally irradiated adult CD45.2 secondary recipients. Multilineage donor-derived engraftment was measured in PB of secondary recipients after 4 months. Limited CD45.1^+^ engraftment (<1% donor chimerism) was observed for secondary recipients transplanted with BM cells from primary recipients that received TS- and TSW-treated 50 LSK progeny cells **(** **Figure 3A** **)**, indicating absence of self-renewing CR-LTR-HSC activity in 50 LSK progeny cells. For animals that received BM from primary recipients grafted with progeny of 100 and 200 TSW-treated LSK progeny, 1/8 and 0/13 were positively repopulated with CD45.1^+^ whereas 3/15 and 5/18 secondary recipients of 100 and 200 LSK progeny cultured with TS were positively repopulated. Moreover, the level of CD45.1^+^ chimerism was lower for secondary grafts from TSW- than TS- treated LSK progeny (both for 100 and 200 cell dose) **(** **Figure 3A** **)**. Importantly, the CRU frequency in secondary recipients of TSW-treated grafts was significantly 6.3-fold lower than TS-treated grafts (p = 0.0314) **(** **Figure 3B** **)**.

Cumulatively, these studies demonstrate therefore that Wnt5a does not enhance, and even impede, maintenance of CR-LTR-HSC, when cultured in serum-free, stroma-free conditions.

**Figure 4:**
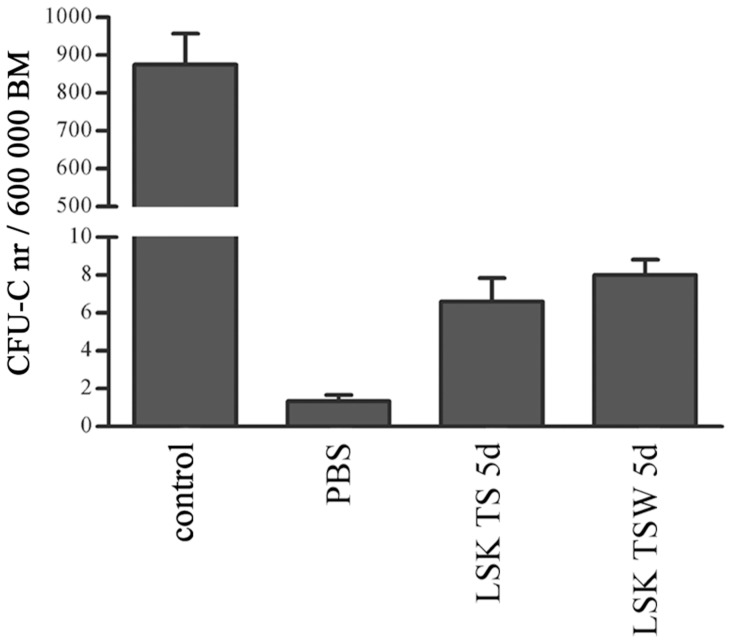
*In vivo* homing of LSK cell progeny cultured *ex vivo* with and without Wnt5a. *In vivo* homing of LSK cell progeny cultured for 5 days with TPO and SCF alone (TS) or with Wnt5a (TSW) was measured by scoring CFU-C in 6×10^5^ BM cells, harvested 16 hrs after infusion of 25,000 LSK cell progeny into lethally irradiated mice. BM cells harvested from nonirradiated mice served in this homing assay as a positive control readout for CFU-C (defined as control in the graph); PBS = BM from mice that were lethally irradiated and injected i.v. with PBS served as negative control; LSK TS 5d = BM from mice that were lethally irradiated and injected with 5days TS-treated LSK progeny cells; LSK TSW 5d = BM from mice that were lethally irradiated and injected with 5 days TSW-treated LSK progeny cells. The data represent here the mean of 3 experiments with SEM of CFU-C frequency in the BM.

As we did not find a significant decrease in total cell or CFU-C expansion, we hypothesized that the lower levels of CD45.1 chimerism in animals that received TSW treated LSK progeny might be caused by decreased homing. We therefore performed *in vivo* homing experiments, using a protocol adapted from Szilvassy *et al.*, [17]. 2.5×10^4^ LSK progeny cells from TS-containing cultures with or without Wnt5a were injected i.v. into lethally irradiated mice. BM was harvested from each recipient 16 hrs after transplantation and CFU-C per 6×10^5^ BM cells were enumerated as a measure of donor-derived progenitor cells that had homed into the BM. We did not observe significant differences in the total CFU-C number (p = 0.38) between animals grafted with LSK progeny from TS and TSW cultures (**Figure 4**).

## Discussion

Here, we demonstrate that addition of Wnt5a to feeder- and serum-free cultures of LSK cells, also supplemented with SCF and TPO, does not improve but impedes, the engraftment potential of HSC compared with cultures supplemented with SCF and TPO. This cannot be explained by decreased survival *in vitro*. In addition, our data obtained on donor-derived committed progenitors showing unaffected *in vivo* homing upon Wnt5a treatment, although not strictly representing HSCs homing potential, may suggest that observed Wnt5a effect on HSC activity could not be explained either by decreased homing. As has also been noted for studies evaluating the effect of another Wnt, Wnt3a, we hypothesize that differences between the current study and other published reports [7], [14], [16] may be due to differences in culture conditions used to test the effect of Wnt5a.

During development, Wnt5a is expressed in different ontogenically hematopoietic sites. However, expression of the putative receptors for Wnt5a, namely Lrp5 and Fzd4, appears highest during the first steps of hematopoietic commitment from embryonic stem cells, suggesting that Wnt5a may function in development and to a lesser degree in adult hematopoiesis [5]. Few studies have addressed a possible role of Wnt5a in postnatal murine hematopoiesis [7], [14]. Nemeth *et al.,* reported that Wnt5a, but not Wnt3a, added alone to stroma-free, serum-free cultures of lineage^−/^c-Kit^+^/Sca-1^+^/IL-7R^-^ (LSKI) could improve the repopulating ability of LSKI cells at 4 months in primary adult recipients. However, in that study the effect of Wnt5a on LTR-HSC activity was not examined in secondary recipients where we found the greatest decrease in the engraftment and expansion for LSK progeny of Wnt5a treated cells.

Malhotra *et al.,* demonstrated that culture of Lin^−/^RAG-1-GFP^−/^c-Kit^+^/Sca-1^+^/Thy1.1^low^ cells on OP9 feeders overexpressing Wnt5a, but not Wnt3a, enhances commitment to the B-lineage *in vitro* [14]. Our group demonstrated that the FL-derived EL08-1D2 cell line, which expresses low levels of Wnt5a, does not support maintenance of LTR-HSC, whereas the UG26-1B6 feeder, which expresses high levels of Wnt5a, supports LTR-HSC in non-contact cultures [16]. Addition of soluble Wnt5a to non-contact EL08-1D2 cultures restored the ability of this feeder to support maintenance of LTR-HSC, suggesting a role for Wnt5a in postnatal HSC biology. However, that study did not address if Wnt5a affects the maintenance of HSC cell autonomously. Therefore, here we tested the effect of Wnt5a on HSC activity using a serum- and stroma-free culture system, still complemented with 2 other growth factors known to maintain HSC, namely SCF and TPO.

In contrast to the studies by Nemeth *et al.,* we found that addition of Wnt5a to stroma- and serum-free cultures did not enhance the repopulation potential of LSK progeny, but that repopulation and expansion of LTR-HSC appeared to be decreased. Also in contrast to Nemeth *et al.,* we found that cell expansion and generation of CFU-C from LSK progeny cultured for 5 days with Wnt5a was unaffected. Whether differences in either cell purity (LSK versus LSKI cells), culture conditions (with TPO and SCF versus without), and/or Wnt5a concentration (100 ng/ml versus 500 ng/ml) are responsible for the differences observed is unclear. As it has recently been shown that HSC repopulating activity is regulated by Wnt signalling in a dose-dependent manner [21], a 5-fold higher concentration of Wnt5a used in the study by Nemeth *et al.,* may be one of the potential explanations. Further, the effect of Wnt5a on LTR-HSC activity was not examined in secondary recipients by Nemeth *et al.* where we observed the most decrease in the engraftment and CRU frequency of Wnt5a-treated LSK progeny compared to control TS-treated LSK cultures. Nevertheless, the effect of Wnt5a on homing of cells to the BM was similar in both studies.

Malhotra *et al.,* demonstrated that Wnt5a induces B-lymphoid commitment *in vitro* [14]. We here show that there is no *in vivo* preferential B-lymphoid engraftment. Again, differences in culture conditions may be responsible for these differing effects. Malhotra *et al.,* cultured HSC on OP9 feeders overexpressing Wnt5a, whereas in the current study cells were cultured without feeders and serum. In Buckley *et al.,* we also demonstrated that addition of Wnt5a to EL08-1D2 based cocultures changed preferential T-lymphoid engraftment seen without Wnt5a to higher B-lymphoid engraftment, when Wnt5a was added. These results suggest that the effect of Wnt5a on B-lymphoid lineage commitment may not be Wnt5a autonomously, but may be co-determined by other signals elaborated by stromal cells.

In conclusion, our data indicate that Wnt5a, when used in serum- and stroma-free cultures also containing SCF and TPO does not enhance adult murine LTR-HSC maintenance and expansion. The differences seen in this study with other published studies further highlight the impact of additional signals and growth factors produced by stromal cells on Wnt5a-mediated effects on HSC.

The stroma-free and serum-free model used in this study to evaluate the role of Wnt5a in regulation of adult HSC repopulating activity is clinically a more relevant culture system, compared to previously published studies wherein either serum or stromal cells were used. In addition, omission of stromal cells and serum allows the examination of the direct effects of Wnt5a on HSC. Our studies strongly suggest that the goal to induce *ex vivo* amplification of transplantable adult HSC using defined factors will not benefit from the addition of Wnt5a alone.

## Supporting Information

File S1 Detailed description of materials and methods applied in the study.
(DOC)
