# Supplementary material for: Wnt5a Does Not Support Hematopoiesis in Stroma-Free, Serum-Free Cultures
Source: PLoS One. 2013 Jan 14;8(1):e53669. doi: 10.1371/journal.pone.0053669 (PMC3544900; doi:10.1371/journal.pone.0053669)
Supplement: File S1 — Detailed description of materials and methods applied in the study. (DOC) [file pone.0053669.s001.doc]

**File S1**

This supplementary file contains a detailed description of materials and methods applied in the study.

*Isolation of LSK cells*

Tibias and femurs were taken from mice, and bone marrow flushed using PBS. Lineage negative (Lin-) cells were isolated using the Lineage Negative Selection Kit (Stem Cell Technologies) according to the manufacturer’s protocol. Lin^-^ cells were stained with antibodies against: lineage markers (APC mouse lineage antibody cocktail; BD Biosciences), Sca-1 PE conjugated (clone D7; eBioscience) and c-kit (CD117) FITC-conjugated (clone 2B8). For each antibody fluorochrome-matched appropriated isotype controls were applied. LSK cells were then sorted using a FacsVantage (BD Biosciences, San Jose, CA).

*CFU-C Assays,* were done using standard culture conditions. Progeny of 50 LSK progeny cultured for 5 days with TPO and SCF or with TPO, SCF and Wnt5a were plated in methylcellulose medium (M3234, Stem Cell Technologies), containing 20ng/ml rm SCF, 10ng/ml rmIL-3, 10ng/ml rmIL-6 (all from R&D Systems), and 3U/ml rh Epo (Centocor BV, Leiden, The Netherlands), and incubated at 37°C and 5% CO_2_. Total number of CFU was scored at day 7 of CFU-C culture.

*Competitive Repopulation Studies*

Lethally irradiated CD45.2 recipient mice (8-12 weeks old) were transplanted with TS or TSW treated progeny of 50, 100 and 200 LSK cells from 8-12 weeks old CD45.1 along with 1x10^5^ recipient’s bone marrow cells into adult lethally irradiated CD45.2 recipient mice (8-12 weeks old). Four months following transplantation, recipients were bled. Donor-derived engraftment was measured in peripheral blood, including multilineage engraftment analysis (B- and T- lymphoid, myeloid) using the following antibodies against: CD45.1 FITC-conjugated (clone A20; BD Pharmingen), CD45.2 PerCp-Cy5.5conjugated (clone 104; BD Pharmingen), B220 APC-conjugated (clone RA3-6B2; eBioscience), Mac-1 PE-conjugated (clone M1/70; eBioscience), Gr-1PE-conjugated (clone RB6-8C5; eBioscience), CD4 PE-conjugated (clone GK1.5; BD Pharmingen) and CD8 PE-conjugated (clone 53-6.7; BD Pharmingen). Flow cytometry analysis was performed on a FACSCanto (BD Biosciences, San Jose, CA). Positive repopulation was defined as ≥ 1% donor-derived cells contributing to all 3 hematopoietic lineages (T- and B- lymphoid, myeloid) in the PB. For secondary transplantations 1x10^6^ of total BM cells from positively repopulated primary recipients was injected i.v. into 3-5 lethally irradiated adult CD45.2 secondary recipient mice.
